# The Effects of Restricted Peripheral Field-of-View on Spatial Learning while Navigating

**DOI:** 10.1371/journal.pone.0163785

**Published:** 2016-10-19

**Authors:** Erica M. Barhorst-Cates, Kristina M. Rand, Sarah H. Creem-Regehr

**Affiliations:** Department of Psychology, University of Utah, Salt Lake City, Utah, United States of America; University of Nottingham, UNITED KINGDOM

## Abstract

Recent work with simulated reductions in visual acuity and contrast sensitivity has found decrements in survey spatial learning as well as increased attentional demands when navigating, compared to performance with normal vision. Given these findings, and previous work showing that peripheral field loss has been associated with impaired mobility and spatial memory for room-sized spaces, we investigated the role of peripheral vision during navigation using a large-scale spatial learning paradigm. First, we aimed to establish the magnitude of spatial memory errors at different levels of field restriction. Second, we tested the hypothesis that navigation under these different levels of restriction would use additional attentional resources. Normally sighted participants walked on novel real-world paths wearing goggles that restricted the field-of-view (FOV) to severe (15°, 10°, 4°, or 0°) or mild angles (60°) and then pointed to remembered target locations using a verbal reporting measure. They completed a concurrent auditory reaction time task throughout each path to measure cognitive load. Only the most severe restrictions (4° and blindfolded) showed impairment in pointing error compared to the mild restriction (within-subjects). The 10° and 4° conditions also showed an increase in reaction time on the secondary attention task, suggesting that navigating with these extreme peripheral field restrictions demands the use of limited cognitive resources. This comparison of different levels of field restriction suggests that although peripheral field loss requires the actor to use more attentional resources while navigating starting at a less extreme level (10°), spatial memory is not negatively affected until the restriction is very severe (4°). These results have implications for understanding of the mechanisms underlying spatial learning during navigation and the approaches that may be taken to develop assistance for navigation with visual impairment.

## Introduction

Spatial learning during navigation of novel spaces is especially challenging for those with severe vision loss. We have argued that challenges occur not only because there is less visual information that can be relied upon by the viewer, but also because navigating with reduced visual information requires dedication of attentional resources for safe mobility [1]. Our previous work showed that navigation with simulated severely degraded acuity and contrast sensitivity led to impaired spatial learning compared to normal viewing conditions. However, little is known about the influence of another very relevant type of vision loss—reduced peripheral-field—on comparable navigation tasks.

Research on peripheral field-of-view (FOV) loss has primarily examined effects on mobility tasks such as stability and obstacle avoidance (e.g., [2]), memory for static displays [3], and locomotion-based memory or spatial updating tasks in smaller scale (room-sized) real and virtual environments [4–6]. In general, previous work suggests that peripheral field is important for an understanding of small-scale spatial layout, but how reduced field influences spatial learning during navigation of novel large-scale spaces is unknown. We focus on two primary questions in a series of four studies requiring navigation in normally sighted individuals with simulated reduced peripheral field. First, does field loss impair spatial learning while navigating, and if so, is there a clear critical extent of field loss under which spatial learning is impaired? Second, does navigation with simulated restricted peripheral field demand additional cognitive resources?

The current set of studies uses a spatial learning and memory paradigm that involves active navigation through novel paths. Thus, it is important to consider what we know about the effects of reduced peripheral field on both the *mobility* and *spatial memory* components of navigation. The importance of peripheral field for successful mobility has been established with tasks assessing both the speed and accuracy of walking through novel paths [2, 7, 8]. For example, Turano et al. [2] observed a significant correlation between the degree of the visual field and performance on two mobility tasks with simulated peripheral field loss—namely, the greater the peripheral field loss, the slower the walking speed and the greater number of “bumps” into obstacles. The authors suggest that visual field may contribute a unique and necessary component to mobility, above and beyond both visual acuity and contrast sensitivity (see also [7], for similar work with clinical low vision participants). Other research shows that as vertical peripheral field becomes more restricted, people adapt their mobility by shifting strategies. Individuals slow down walking speed and take wider turns to avoid obstacles [9] or increase toe clearance, step length, and even downward-focused head movements when the task requires stepping over obstacles [10]. This work suggests that people’s behavior during obstacle avoidance changes not as a result of the lack of visual information itself, but rather as a response to the demands on their safety in the environment.

Beyond basic mobility goals of path following and obstacle avoidance, other research has tested effects of reduced peripheral field on spatial memory for configurations of objects in small-scale spaces such as rooms, where all targets can be seen from a single viewing location. This research shows that peripheral field is important for providing a global spatial framework that helps to direct visual search in order to encode and remember individual spatial locations accurately. For example, Yamamoto and Philbeck [3] examined memory for spatial layout from a single static viewpoint in normally sighted people with simulated field loss. They found that viewing with a very restricted FOV (3–5°) resulted in more memory distortions than viewing the layout with a wide FOV, and attributed the increased memory errors specifically to lack of opportunity to direct eye movements to scan for targets. They suggest that eye movements, more than head movements, may be what contribute to accurate and efficient spatial learning, since eye movements allow the viewer to quickly detect and perceive a wide area of space.

Using an immersive virtual environment, Fortenbaugh, Hicks, Hao, and Turano [11] suggest that reduced FOV impairs distance perception, critical to learning and understanding spatial environments. This study examined memory for previously seen layouts of objects when viewed with FOV restrictions of 40, 20, 10, or 0 degrees. In testing, the visible targets were removed and the participants were instructed to walk to the remembered location of each target. The findings showed a significant negative relationship between peripheral field restriction and spatial location error; error increased with decrease in peripheral field. They also varied whether participants viewed the targets from a single stationary position or walked to the targets, and found increased compression of distance when participants remained stationary. This study, as well as a related study with individuals with Retinitis Pigmentosa (RP) who have clinical peripheral field loss [5], suggests that spatial memory errors with reduced FOV may result from errors in distance perception, but that these errors may be reduced when active movement to targets is allowed.

The finding that active motion through an environment improves spatial perception for those with FOV loss also introduces the question of how optic flow information that occurs with observer movement influences spatial learning. There is a body of work showing the importance of optical flow on perception of self-motion, demonstrating illusory perception of self-motion in the stationary observer with either peripheral or central field stimulation [12–14]. It has also been shown that optic flow alone can be used to determine heading direction and travel distance [15, 16]. With actual observer movement, studies of perceptual-motor learning show the importance of optic flow for calibrating spatial updating [17, 18]. Given these findings, it is likely that the limited access to optic flow information resulting from restricted FOV impairs the ability to encode spatial information through navigation. This finding is supported by Fortenbaugh et al. [4], who demonstrated that although self-motion through an environment did reduce distance underestimation biases for those with simulated reduced FOV, participants still showed increased underestimation as FOV restrictions increased.

An area that has received less attention with respect to visual degradation, specifically peripheral field loss, is the impact of vision loss on the ability to acquire spatial knowledge during navigation through large-scale environments. Of interest in the current study is how peripheral field loss influences the formation of *cognitive maps*, or survey-based representations of spaces [19]. Cognitive maps are generally considered to require an allocentric understanding of space, deviating from route-based information and small-scale spatial encoding that can be achieved from a single viewpoint. This distinction is important not only because cognitive maps provide a more flexible understanding of a spatial environment, but also because of the effort required in forming a cognitive map through navigation in a novel space. A robust finding in spatial memory research is that large-scale cognitive map formation is an attention-demanding task, placing strong demands on cognitive resources. The requirement of attention and cognitive resources during cognitive map formation is evident by the fact that cognitive maps are impaired by intent to learn [20] and, most commonly, disrupted by divided attention [21–24].

We argue the need for attentional resources for spatial learning is particularly relevant for those with visual impairments due to competing demands on these resources that emerge from several factors. First, consider independent mobility itself. All individuals must pay attention to their environment while navigating to ensure they do not collide with objects, maintain a straight path, and otherwise keep safe while walking. This attentional requirement is typically minimal for those with normal vision unless the environment is potentially less safe for walking, such as walking on a slippery surface or crossing a busy street. Those with visual impairments likely face additional *mobility monitoring* demands, or the cognitive demands required to walk safely through a space due to reduced access to the visual environment [1]. In addition to monitoring safe mobility during locomotion, individuals with severe vision loss may also experience additional demands on their cognitive resources, such as those required for spatial temporal integration, a process that may be extra challenging for those who cannot view the entire moving scene at once. This process in a restricted peripheral vision state may require the viewer to expend more cognitive effort to gain the same information.

To assess the potential of additional attentional resources needed while navigating with reduced FOV compared to a wide FOV, we adopted a method that deviates from the commonly used dual task paradigm. Instead of assessing how the addition of dual tasks affects spatial learning performance, we used an auditory reaction time task that provides a sensitive measure of available cognitive resources while not significantly disrupting concurrent task performance. Doing so allows us to examine the impact of mobility monitoring on attentional resources while individuals are navigating and attempting to form cognitive maps. Rand et al. [1] took this approach in a navigation and spatial learning task performed by normally sighted participants wearing goggles that simulated visual impairments of degraded acuity and contrast sensitivity, resulting in severely blurred vision with a relatively intact field of view (60°). Using the auditory reaction time task, they demonstrated that navigation with severely degraded acuity and contrast sensitivity uses increased attentional resources to ensure safe mobility, and that these attentional demands accounted for some of the spatial memory error. Given these effects for reduced acuity and contrast, we predicted that navigating with reduced peripheral field—known to affect mobility even more than reduced acuity—would lead to similar increased attentional demands.

Our studies were motivated by two aims related to unknown effects of reduced peripheral field on memory for spatial layout learned while navigating on novel paths. We aimed first to establish the magnitude of spatial memory errors at different levels of field restriction. Testing normally sighted participants in a virtual environment, Hassan, Hicks, Lei, and Turano [25] found that the critical field size for efficient navigation (defined by walking speed and obstacle avoidance) depends on the image contrast levels of the environment, with critical points of 32.1° for low image contrast levels, 18.4° for medium, and 10.9° for high. In a study of clinical low vision patients, Fortenbaugh, Hicks, and Turano [5] observed a significant negative relationship between peripheral field loss and placement error for remembered object locations. As the field-of-view of clinical patients decreased from 20° to 10° and narrower, the mean placement error for the remembered objects increased. These findings directed us to begin with a moderately reduced FOV of 15° in Experiment 1, reduced to 10° in Experiment 2, and reduced further to 4° in Experiment 3. Finally, Experiment 4 implemented the navigation task completely without vision to test the extent to which visual information was even necessary to succeed on the learning task given the non-visual body-based (proprioceptive and vestibular) information available from active navigation. We refer to each of these conditions as experiments because they involved separate groups of people and included a within-subjects comparison to a large FOV of 60° (referred to as vision condition), but we present them together because they followed the same methods and procedure. We predicted greater errors in spatial memory in the reduced FOV conditions compared to the 60° control condition, and examined the possibility that the difference in error would increase as the restriction became more severe.

Our second aim was to ask whether navigation under these different levels of restriction would place a greater tax on available cognitive resources. To test this question, we included a secondary auditory task performed concurrently with the navigation. We expected that demands for monitoring safe mobility would increase with FOV restriction and blind conditions compared to a wide FOV following the rationale of Rand et al. [1]. In addition, cognitive demands could increase as a result of differences in processes associated with restricted field such as effects on the spatial-temporal integration required in spatial learning while navigating. Thus, here, we predicted that cognitive resource competition during navigation with restricted FOV or no vision at all, would be reflected in slower response times on the secondary auditory task compared to the wide FOV condition. We additionally predicted that this increase in cognitive load due to FOV restrictions would, in part, explain observed differences in spatial learning, consistent with our findings with reduced contrast sensitivity and acuity.

## Method

### Participants

One hundred and sixteen participants completed the study (75 female). Ages ranged from 18 to 50 years (M = 22.8, SD = 6.02). Thirty-two participants completed Experiment 1 (23 female). Data from one participant was removed because of a recording error during the attention task. Twenty-eight participants completed Experiment 2 (17 females). Twenty-eight participants completed Experiment 3 (16 female). Twenty-eight participants completed Experiment 4 (19 female).

Participants were recruited for all experiments from either the University of Utah psychology participant pool or from the broader community. All participants gave written informed consent with procedures approved by the University of Utah’s Institutional Review Board. University of Utah students were compensated with partial course credit and participants from the broader community were paid $10 as compensation for their time. All participants had normal or corrected to normal vision and walked without impairment. None performed in more than one experiment.

### Field-of-View Restriction

In each experiment, all participants wore two sets of goggles: a narrow and wide FOV. Both were welding goggles with the original plastic lenses removed. The FOVs of each set of goggles were restricted in the dominant eye using black cardstock paper with the appropriate-sized hole cut out of the center of the circle. The front face of the goggles (where the paper was placed) was approximately 4 cm from the viewer’s eye, although the exact distance between the eye and aperture varied slightly between participants due to face shape and goggle fit. To achieve a monocular field-of-view around 4° for Experiment 3, the goggles were updated with a truncated cone with a length of 2.5-inches on the covering of the dominant eye. The aperture was cut out of the far end of the cone. The hole for the wide goggles was 42 mm in diameter. The holes for the 15°, 10°, and 4° FOV goggles were 7 mm, 3 mm, and 1 mm in diameter, respectively. The non-dominant eye was covered completely. As a manipulation check of actual restricted FOV, we conducted an aperture-viewing test. During training, participants wore each set of goggles and were instructed to walk toward a black circle constructed at a diameter that matched the intended FOV as viewed through the goggles at an approximate distance. They were instructed to stop as soon as the circle filled their entire peripheral vision. The black circle on the wall was placed at the participant’s eye height. The distance from the target at which the participant stopped (i.e., the distance at which the circle “filled the participant’s vision”) was used to calculate the degree of FOV that the participant perceived. Although the intended FOVs were 15°, 10°, and 4° for the restricted conditions and 60° for the control condition in each experiment, the measured FOVs averaged 17.8° (*SD* = 4.8), 11.14° (*SD* = 1.3), and 4.60° (*SD* = .62), respectively, and 67.9° (*SD* = 8.7) across the 4 control conditions. While the aperture size was determined based on the intended FOV and the approximate distance of the goggles from the eyes, there was some variability between participants, likely due to the way the goggles fit each individual’s head. For Experiment 4, the goggles were adjusted to simulate total blindness. To do this, the goggles were covered with an opaque screen covered with thick black electrical tape.

### Procedure

All experiments took place in the Merrill Engineering Building on the University of Utah campus, a building that was novel to the majority of our participants. A familiarity rating given at the end of the experiment showed that across all experiments, 91.4% rated the environment on a 7-point Likert scale as 6 or 7 (very unfamiliar). As a check that familiarity did not influence performance, those participants who reported any type of familiarity with the building (6 or less) were removed from the data and analyses were run with and without them. Excluding participants who reported familiarity with the building did not change the results, so they were included in all analyses.

Upon arrival, participants signed a consent form and filled out demographics information. Participants were tested for normal or corrected-to-normal visual acuity using the Lighthouse distance visual acuity chart and tested for the dominant eye. They were then trained on the secondary, auditory reaction time task [26], intended to be a measure of cognitive load [27]. Participants listened through wireless headphones to a series of randomly generated tones that occurred every 1–6 seconds. They were instructed to respond to each tone by clicking a cordless mouse as quickly as possible after hearing the tone. Tones were generated and responses were recorded on a laptop carried by a second experimenter throughout the experiment. During the practice, the volume of the tones was adjusted to suit the participant’s comfort. During the experiment, this task was performed concurrently with the spatial learning/navigation task described below.

**Fig 1:**
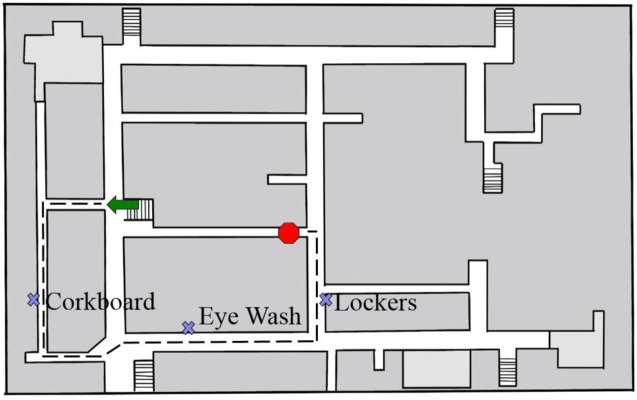
Example walking path with 3 landmarks.

All participants walked a total of 4 paths in the Merrill Engineering Building at the University of Utah: 2 paths with restricted field of the dominant eye (the degree of restriction was specific to each experiment), and 2 paths with the wide field vision condition (the FOV that resulted from wearing the control goggles, ~60°). The within-subjects manipulation of vision condition was essential in controlling for known individual differences in spatial learning [28], which are often large. With this design we could evaluate within each subject the relative difference between wide and restricted FOV even if individuals varied on the magnitude of their overall spatial learning error. Each path contained 3 distinct landmarks, so participants encountered 6 landmarks in the narrow FOV condition and 6 landmarks in the wide FOV condition (see Fig 1). Each path had 4 turns, and ranged from 109–121 meters in length, taking approximately 3 minutes to complete. Each of the four paths was unique, such that there was no crossover from one path to another and the participant was not exposed to the same paths or landmarks more than once. The order of the vision condition was manipulated between subjects and counterbalanced, such that half of participants completed the paths in a restricted-normal-restricted-normal order, and the other half of participants completed the paths in a normal-restricted-normal-restricted order. Participants walked along the paths following verbal instructions from the experimenter (e.g., walk forward, stop, turn right), who maintained position on the side opposite the participant’s dominant hand and slightly behind the participant, without touching the participant. The experimenter assured each participant that she would do her best to maintain the safety of the participant (i.e., not letting him or her run into walls or objects, etc.). The experimenter stopped the participant at each landmark and verbally described the location of the landmark (e.g., “Stop here. On your left is a water fountain”). During practice, participants were encouraged to look at each target object while stopped next to it. After a three-second pause, the experimenter encouraged the participant to continue walking. Participants were told during training that their memory for the objects would be tested in a random order, not necessarily in the order in which they encountered the objects on the path. Although the participants were not aware of which objects on the path would be target landmarks, participants were able to view the hallway as they walked and were instructed to look around. To reinforce the importance of turning one’s head to look around, during part of the training participants were told to read the numbers off of doors in the hallway as they walked. As such, participants were able to see the landmarks as they approached them, if they were looking that way. However it was not possible for participants to see more than one landmark at a time, even during approach.

**Fig 2:**
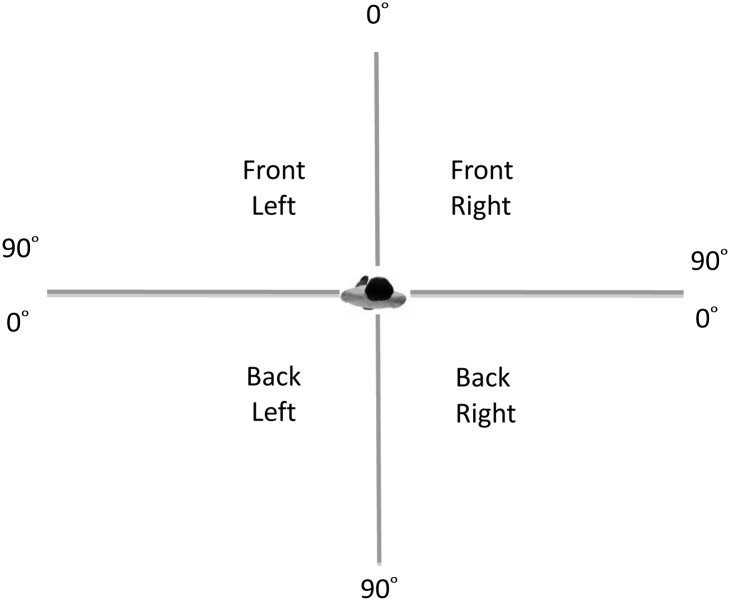
Illustration of the Degree-Quadrant Pointing Task. Depiction of the task developed by Philbeck, Sargent, Arthur, and Dopkins [29].

We tested spatial memory using a verbal-pointing measure [29]. At the end of each path, participants indicated the location of each landmark as probed by the experimenter (in a random order) by pointing directly to the landmark and verbally describing each landmark’s location. Participants were instructed to remain in the orientation established at the end of the path, and point directly to the landmark from the final location and in the same facing direction, as if they could see straight through the walls right to the landmark. To describe the pointing direction, participants were trained to use a two-step verbal response. Participants were instructed to first divide the 360 degrees of space surrounding them into four quadrants (e.g., front-left, front-right, back-left, or back-right). Next, participants were trained to provide a degree from 0–90 within each quadrant that specifically indicated the direction to the remembered target location (see Fig 2). This quadrant/degree response was converted into an absolute error value for each landmark. Although the verbal description of pointing was used as the recorded dependent measure, we used the physical pointing as a check to ensure that participants were correctly reporting their intended direction.

Participants were then asked to rate their level of self-reported anxiety on the Subjective Units of Distress Scale [30], on a scale from 0 to 100, for 1) the path on average, 2) when turning corners, and 3) when encountering people. Participants were specifically instructed to report their anxiety as it related to their safety while navigating. Finally, participants completed one minute of the auditory reaction time task while standing stationary to establish a baseline reaction time for each path. This procedure was repeated for the remaining 3 paths. After completing the 4 paths, participants filled out a Memory Anxiety Questionnaire [31] and the Santa Barbara Sense of Direction Scale [32]. At the end of the experiment, they were then debriefed, thanked, and dismissed. Scores on the Santa Barbara Sense of Direction scale and the Memory Anxiety questionnaire were not significant predictors of spatial memory performance on any of the experiments, and were thus dropped from further analyses.

## Results

### Spatial Memory

**Fig 3:**
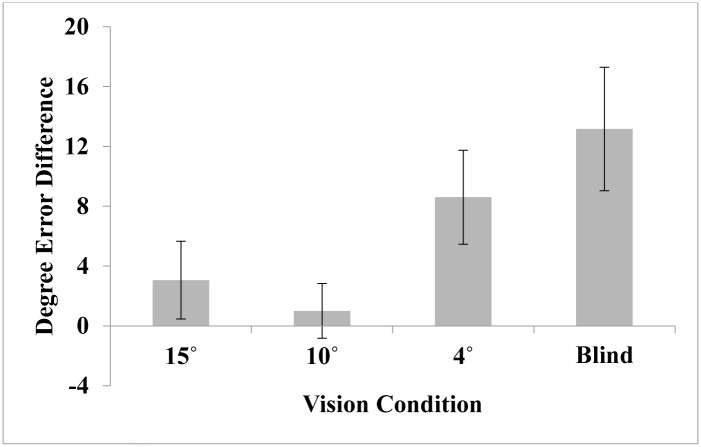
Difference in pointing error between the wide and narrow conditions for Experiments 1–4. The difference score was calculated by subtracting narrow—wide error for each participant. The average difference for each experiment is plotted. Error bars represent +/- 1 standard error (*SE*) of the mean.

Absolute error was calculated as the absolute value of the difference between the pointing vector and the correct vector. The difference score was calculated for each target and averaged across the 6 trials for each FOV condition. We set a cut-off criterion for below-chance performance as 90 degrees of average error in either vision condition [1]. No participants in the current series of studies met this criterion and all were included in the analyses. Four participants across the four studies performed more than 3 standard deviation values above the mean in either pointing error or reaction time. Their removal as outliers does not change the statistical outcomes. They are included in the reported analyses. A mixed-design analysis of variance (ANOVA) with vision condition as a within-subjects variable and vision order as a between-subjects variable was conducted separately on absolute error for each experiment. Overall, as described in the results below, spatial memory was not affected by the narrow restricted field until the field restriction was very extreme (4° and blind). Performance at 15° and 10° was not different than performance in the control condition in each case (see Fig 3 for difference scores and Table 1 for mean pointing error for each experiment and condition).

**Table 1:** Pointing error results from Experiments 1–4.

			Narrow		Wide		
Experiment	FOV	*n*	*M*	*SE*	*M*	*SE*	*η*_*p*_^*2*^
**1**	15°	31	29.18°	2.87°	26.24°	2.94°	.042
**2**	10°	28	20.75°	1.64°	19.74°	1.66°	.015
**3**	4°	28	30.93°	3.54°	22.21°	1.28°	.216*
**4**	0°	28	39.581°	4.11°	26.42°	2.59°	.273**

*p < .05,

**p < .01

For Experiment 1, in contrast to our initial predictions, we did not find evidence in support of a difference between vision conditions in average absolute error on the memory task. The ANOVA revealed that absolute error for the 15° FOV condition (*M* = 29.18°, *SE* = 2.87°) did not differ significantly from error for the 60° FOV condition (*M* = 26.24°, *SE* = 2.94°), *F*(1, 29) = 1.28, *p* = .268, *η*_*p*_^*2*^ = .042; there were no effects of vision order (*p* > .1).

Experiment 2 also surprisingly indicated no overall difference in average absolute pointing error between 10° and 60° FOV conditions, despite the more extreme restriction. Error for the 10° FOV condition (*M* = 20.75°, *SE* = 1.64°) did not differ significantly from error for the 60° FOV condition (*M* = 19.74°, *SE* = 1.66°), *F*(1, 26) = .384, *p* = .541, *η*_*p*_^*2*^ = .015. There was, however, a significant vision condition x vision order interaction, *F*(1, 26) = 8.287, *p* < .01, *η*_*p*_^*2*^ = .242. Post hoc ANOVAs comparing vision condition for each group order separately revealed more error for the 10° FOV condition (*M* = 21.66, *SE* = 2.17) compared to the 60° condition (*M* = 15.99, *SE* = 1.54) only for the group of participants who performed the 10° condition first, *F*(1, 13) = 6.44, *p* < .05, *η*_*p*_^*2*^ = .331. There was no difference between vision conditions for those who performed the 60° condition first, *F*(1, 13) = 2.43, *p* = .143, *η*_*p*_^*2*^ = .158. While these results may suggest some advantage of performing the task first with a wider FOV, it is somewhat difficult to interpret order effects because the conditions alternated and were not blocked (i.e., these participants performed the tasks with the order: restricted, wide, restricted, wide).

However, in Experiment 3, when the restriction was reduced to 4°, we found that absolute pointing error on the memory task increased with the narrow restricted FOV. Pointing error for the 4° FOV condition (*M* = 30.93°, *SE* = 3.54°) was significantly greater, *F*(1, 26) = 7.16, *p* < .05, *η*_*p*_^*2*^ = .216, than error for the 60° FOV condition (*M* = 22.21°, *SE* = 1.28°). Vision order showed no effects (*p* >.3).

Finally, in Experiment 4, as with the 4° FOV, absolute pointing error after learning while blindfolded was greater compared to the wide FOV condition. Pointing error for the blind condition (*M* = 39.58°, *SE* = 4.11°) differed significantly, *F*(1, 26) = 9.781, *p* < .01, *η*_*p*_^*2*^ = .273, from error for the 60° FOV condition (*M* = 26.42°, *SE* = 2.59°). There were no effects of vision order (*p* > .1).

Given the unexpected apparent difference in absolute error for the 60° FOV (control) condition across experiments, we investigated this further with a univariate ANOVA on absolute pointing error for only the 60° condition, including experiment as a between-subject factor. There was no significant main effect of experiment, *F*(3,111) = 1.92, *p* = .13, *η*_*p*_^*2*^ = .049, suggesting that performance in the control condition did not differ significantly across the 4 experiments. However, planned contrasts comparing each experiment to Experiment 1 did show that error was marginally lower for Experiment 2 compared to Experiment 1 (*Mean difference* = 6.40, *SE* = 3.23, *p* = .05), but neither Experiment 3 (*p* < .24) nor Experiment 4 (*p* < .93) differed from Experiment 1. These results suggest the importance of examining individual differences factors that could contribute to baseline error and variability differences and this is further considered in the Discussion.

### Cognitive Load

**Fig 4:**
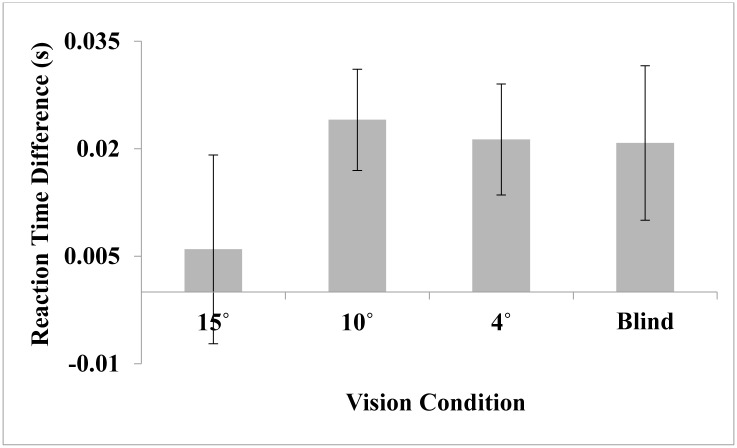
Difference in reaction time between the wide and narrow vision conditions in Experiments 1–4. The difference score was calculated by subtracting narrow error- wide error for each participant. The average difference for each experiment is plotted. Error bars represent +/- 1 standard error (*SE*) of the mean.

Cognitive load was calculated as the average response time to the beeps presented while completing the path (approximately 3 minutes of randomized beeps). A slower response time indicates greater cognitive load [27]. A mixed-design analysis of variance (ANOVA) with vision condition as a within-subjects variable and vision order as a between-subjects variable was conducted separately on reaction time for each experiment. Overall, reaction time was not affected by the field restriction at 15° but was slower in both the 10° and 4° vision conditions compared to the wide control. There was also a trending increase in reaction time in the blind condition (see Fig 4 for difference scores and Table 2 for RT means).

**Table 2:** Reaction time results from Experiments 1–4.

			Narrow		Wide		
Experiment	FOV	*n*	*M*	*SE*	*M*	*SE*	*η*_*p*_^*2*^
**1**	15°	31	.609	.02	.603	.01	.006
**2**	10°	28	.644	.012	.619	.013	.314**
**3**	4°	28	.642	.013	.621	.014	.241**
**4**	0°	28	.618	.016	.597	.014	.122

**p < .01

For Experiment 1, there was no significant difference in reaction time between the 15° condition (*M* = .609, *SE* = .02) and the 60° FOV condition (*M* = .603, *SE* = .01), *F*(1, 29) = .176, *p* = .678, *η*_*p*_^*2*^ = .006 and there were no effects of vision order (*p* > .05). However, in Experiment 2 there was a significantly slower reaction time on the auditory task (*F*(1, 26) = 11.91, *p* < .01, *η*_*p*_^*2*^ = .314) in the 10° condition (*M* = .644, *SE* = .012) compared to the 60° FOV condition (*M* = .619, *SE* = .013). This suggests that participants had a greater cognitive load when navigating with the 10° goggles compared to when navigating with the wide FOV. There were no effects or interactions with vision order on reaction time (*p* > .50). Similarly, in Experiment 3 reaction time was greater (*F*(1, 26) = 8.26, *p* < .01, *η*_*p*_^*2*^ = .241) in the 4° condition (*M* = .642, *SE* = .013) compared to the 60° FOV condition (*M* = .621, *SE* = .014), suggesting that participants faced increased cognitive load when navigating with the 4° goggles. There were no effects of vision order (*p* > .05). Finally, in Experiment 4, there was a marginally significant increase (*F*(1, 26) = 3.612, *p* = .068, *η*_*p*_^*2*^ = .122) in reaction time in the blindfolded condition (*M* = 0.6181, *SE* = .016) compared to the 60° FOV condition (*M* = 0.5973, *SE* = .014). There were no effects of vision order (*p* > .1).

To further explore whether cognitive load accounted for the effects on spatial learning, we created a reaction time differences score (wide versus narrow) and ran the same analyses as above with the RT difference score as a covariate. RT was not a significant covariate in any of the experiments (*p* > .1) and the effect of vision condition was unchanged with the addition of the covariate. While it is difficult to interpret a null result, these results suggest at least that there is a contribution of the visual field restriction to the increase in memory error, above and beyond the difference in cognitive load.

### Anxiety

Our final dependent measure was the Subjective Units of Distress (SUDS) scale. Participants reported their anxiety for each path on average, when turning corners, and when encountering people, after completion of the path. Responses across the three questions were averaged to create an overall SUDS score. A mixed-design analysis of variance (ANOVA) with vision condition as a within-subjects variable and vision order as a between-subjects variable was conducted separately on overall SUDS for each experiment. As detailed below, in all 4 experiments, participants reported significantly more anxiety in the more severe field restriction than in the control (60°) condition (see Table 3).

**Table 3:** Subjective Units of Distress Results from Experiments 1–4.

			Narrow		Wide		
Experiment	FOV	*n*	*M*	*SE*	*M*	*SE*	*η*_*p*_^*2*^
**1**	15°	31	33.02	3.60	20.41	2.82	.564***
**2**	10°	28	34.07	3.52	19.42	2.63	.637***
**3**	4°	28	40.46	3.87	17.54	2.23	.664***
**4**	0°	28	49.17	4.01	19.20	2.76	.735***

***p < .001.

In Experiment 1, navigating with 15° restricted FOV (*M* = 33.02, *SE* = 3.60) was associated with higher anxiety ratings (*F*(1, 29) = 37.49, *p* < .001, *η*_*p*_^*2*^ = .564) than navigating with wide FOV (*M* = 20.41, *SE* = 2.82). In Experiment 2, navigating with 10° restricted FOV (*M* = 34.07, *SE* = 3.52) showed higher anxiety ratings (*F*(1, 26) = 45.578, *p* < .001, *η*_*p*_^*2*^ = .637) than navigating with wide FOV (*M* = 19.42, *SE* = 2.63). For Experiment 3, navigating with 4° restricted FOV (*M* = 40.46, *SE* = 3.87) showed higher anxiety ratings than navigating with wide FOV (*M* = 17.54, *SE* = 2.23), (*F*(1, 26) = 51.30, *p* < .001, *η*_*p*_^*2*^ = .664). Finally, Experiment 4 also showed those navigating without vision (*M* = 49.167, *SE* = 4.013) reported more anxiety (*F*(1, 26) = 72.07, *p* < .001, *η*_*p*_^*2*^ = .735) compared to when navigating with a wide FOV (*M* = 19.196, *SE* = 2.762). There was no effect of vision order in any of the experiments (*ps* >.1).

Taken together, the SUDS results suggest participants experience more anxiety while walking in restricted viewing conditions in all cases. This finding supports the notion that walking with restricted field requires additional mobility monitoring. To further examine the effects of anxiety on spatial learning, we computed a difference score between anxiety reported with the wide goggles and anxiety reported for the restricted field goggles, for each experiment. We correlated this difference with the difference in pointing error (depicted in Fig 3) for each experiment to examine how variations in anxiety relate to spatial learning. There were not significant correlations between SUDS and pointing error for Experiment 1 (*r* = -.023, *p* = .9) or Experiment 2 (*r* = -.047, *p* = .814). This would be expected given no difference in pointing error in those experiments. However, a significant positive correlation emerged between the SUDS difference and the pointing error difference in Experiment 3 (*r* = .47, *p* < .02). For Experiment 4, SUDS difference and pointing error difference were not significantly correlated (*r* = .274, *p* = .159). A limitation of the SUDS as an anxiety measure to consider is that it was self-reported after the completion of each path, allowing the participant to reflect on the path and report his or her feelings at the end. This allowed the participant to process past performance rather than capturing the actual experience of anxiety during the trial. A more sensitive physiological measure of anxiety (i.e., heart rate, skin conductance) could be administered during the task in future research to capture the effects of emotion during the task itself.

## Discussion

Across the four experiments, our results suggest that simulated peripheral field loss has a negative impact on spatial memory for the layout of landmarks learned while navigating along novel paths, but only when that restriction reaches a very severe level (4°). This decrement in spatial memory can be explained by two possible mechanisms that likely contribute simultaneously to the demands of the task. First, increased spatial memory errors may come as a result of limited visual information during encoding. Second, these errors may come as a result of the attentional demands experienced while navigating with impaired vision, both from mobility monitoring and compensation for the visual deficit. Below we discuss our findings as they relate to these mechanisms.

### Visual Mechanisms

Previous research shows that restricting the peripheral field to an extreme level impacts spatial encoding within small-scale spaces. These studies showed that greater field restriction negatively affects viewers’ spatial memory for objects seen from a single viewpoint [3] or those experienced by walking in a single room [4, 6]. As these studies have argued, limited peripheral field negatively affects the viewers’ visual understanding of the global space because the visual restriction requires the viewer to extrapolate more of the environment, potentially resulting in an incomplete representation of the space. Our studies are consistent with this claim. In order to process the scene in a way that is equivalent to how one would with normal vision while wearing the field-restricting goggles, one must incorporate more full-head movements in order to fixate on useful visual information. However, the object locations and inter-object relations in the environment may not be integrated in space and time in the same way as they would with normal vision, which may result in a disconnected or less veridical representation of the global environment. While our task did not involve visual integration of multiple targets within a single room as in previous work, more head movements were still required to perceive the positions of target objects with respect to the surrounding hallway context.

In addition, the field restrictions used in the current studies and their effects on performance relate to the limits of foveal vision. While the 15° and 10° goggles both allow for at least some peripheral vision to be potentially used, the 4° goggles essentially limited the peripheral field entirely. The foveal centralis has a field-of-view of about 5° [33], thus requiring our participants to rely solely on foveal vision rather than on some combination of central and peripheral vision in the 4° condition. As such, it is likely that participants could not use eye movements at all in this condition, impairing the ability to track specific features within the hallway while walking. Furthermore, the 4° condition severely restricts the amount of peripheral optic flow that was experienced during walking. The spatial memory impairment seen in this condition supports previous work on the importance of optic flow for perception of self-motion and its contribution to processes of perception of distance travelled and spatial updating [16].

It is also important to look to the completely blind condition to interpret the role of visual information in our spatial learning task. The results of experiment 4 show significantly impaired learning in the blindfolded compared to wide FOV condition. Given the difficulty of comparing the magnitude of errors across between-subject conditions, we ran one additional study that compared 4° and blind as a within-subject manipulation using the same paradigm as the previous experiments. Here, we found no statistical difference between learning with the 4° restriction and a complete lack of visual information for pointing error (*F*(1, 26) = .653, *p* = .426, *η*_*p*_^*2*^ = .024) or response time (*F*(1, 26) = .612, *p* = .837, *η*_*p*_^*2*^ = .002). Interestingly, the SUDS anxiety report was greater for the blindfolded (*M* = 35.4, *SE* = 3.2) versus 4° condition (*M* = 27.2, *SE* = 2.5; *F*(1, 26) = 16.06, *p* < .001, *η*_*p*_^*2*^ = .382), suggesting that participants had a different subjective experience when some visual information was available. However, the equivalent spatial learning performance suggests that the extremely restricted FOV provided little additional information to guide learning and that participants may have switched to rely more on non-visual body-based cues in both Experiments 3 and 4. Understanding how and when residual vision influences learning during navigation is an especially important goal for future work in this area, given the large and growing population who are severely visually impaired but not blind (nei.nih.gov/eyedata).

### Attention Mechanisms

Our study also involved a second mechanism, namely, the added attentional demands related to navigating with severely restricted field. The studies presented here differ from the previous related research described above because of the nature of the type of large-scale environment used. Many of the previous studies were conducted in single room-sized spaces where configurations of objects could be perceived from a single viewpoint with eye and head movements. In contrast, in our studies involving multiple paths, the spatial configuration could not be learned from a single viewpoint. Rather, participants had to maintain a representation of the spatial configuration while navigating through the space and encountering landmarks from separate viewpoints. To accurately point to remembered targets, participants must integrate locally experienced memory for landmarks into a global representation. Thus, our task adds the additional factor—beyond limited peripheral field—of active self-movement through large environments. Active navigation requires the monitoring of one’s own mobility, both for safety and spatial updating, while walking with reduced visual information. The concept of mobility monitoring is very relevant for peripheral-field loss, as Turano and colleagues [2] as well as Jansen and colleagues [9, 10] observed negative effects of field loss on physical mobility (i.e., less stability and decreased ability to avoid obstacles; greater mobility adaptation). The results of our auditory secondary task showed that the act of navigation demanded more attention with limited peripheral field (10° and lower), supporting previous work that peripheral field is important for mobility.

In addition to mobility monitoring, the field restrictions themselves also likely increase cognitive load more than the same task with no field restriction. First, there is added effort for encoding with a visual restriction (i.e., for spatial-temporal integration). A related factor that is specific to the most extreme restriction tested (4°) is the need to integrate multiple views over space and time based on information from head movements, because eye movements could not be relied on in this condition. Our current experiments cannot separate attentional demands related to processing visual information from those related to safe mobility, but based on the increase in anxiety ratings and increase in RT found in the current study, it is likely that both are contributing factors. Consistent with this idea, there are other related studies that have shown a relationship between increased attentional demands and impaired spatial learning and memory. In a study of individuals with normal vision, the ability to form allocentric mental maps of spatial locations was negatively affected by the addition of a concurrent task [22]. In the context of navigation with visual impairment, Klatzky, Marston, Giudice, Golledge and Loomis [34] found that certain navigation aids actually impaired performance on a subsequent cognitive task (*N*-back) during virtual navigation, rather than helping as originally intended. Those aids that required more cognitive processing (i.e., directional language) impaired performance on the *N*-back task as compared to aids that functioned more at the perceptual level (sounds). Klatzky et al. [34] propose that the language aids were detrimental compared to the sound aids because they increased cognitive load, which had negative effects on task performance. Others have suggested that navigational aids impair spatial memory by increasing divided attention between the navigational assistance and the spatial learning goal [35].

Although the results of our study suggest that individuals navigating with simulated restricted FOV of 10 and 4 degrees do demonstrate a decrease in cognitive load while attempting to learn an environment through navigation, the impact of cognitive demands on spatial learning needs more research. Surprisingly considering the causal findings of Rand et al. [1] of cognitive load on spatial learning with simulated degraded acuity and contrast sensitivity, differences in reaction time between narrow and wide FOVs did not predict differences in spatial learning error between narrow and wide FOVs in the current study. That is, although navigating with restricted FOV does increase cognitive load as revealed by the RT measure, the covariate analysis suggested that this increase does not strongly contribute to the spatial learning error.

### Active navigation and spatial learning

The role of self-movement in spatial learning has been examined in a number of different ways, and while only a few have involved low vision, they are useful in the interpretation of our results. At a basic level of spatial updating, much research supports the claim that active walking through a space facilitates automatic updating of the spatial positions with respect to oneself [36, 37] and that spatial updating and judgments of distances walked can be performed accurately without vision [38]. For navigation tasks that require learning of multiple segments, non-visual body-based feedback from locomotion generally aids in survey spatial learning, although there are mixed findings on the relative contributions of motor, proprioceptive, and vestibular information, and it also has been shown that attention plays an important role (see [39] for an extensive review). In the context of simulated low vision, active walking to targets with 10° FOV reduced the bias to compress distance compared to static viewing, but still, increasing biases were seen as field restriction increased [4]. Furthermore, spatial memory ability in clinical low vision populations may be dependent on previous experience with the relationship between visual and body-based information for self-movement, as some have shown that individuals with early-onset field loss showed more spatial memory error after navigating than late-onset or congenitally blind individuals [40]. Together this research suggests that although a wealth of spatial knowledge can be obtained through body-based information, vision-based information that is lacking for those with peripheral restrictions provides additional information that can assist with spatial understanding. In the current studies, it is likely that our participants used body-based information from active navigation to assist in learning the spatial layout in the current tasks, but the role of visual information is still apparent from the increased spatial memory errors at the most severe restrictions (4° and blindfolded).

It is important to consider the possibility that individual differences that were not measured could have contributed to both within- and between-experiment effects in our current set of studies. An unexpected finding was the trend of lower error for the 60° (control) FOV in Experiment 2 when compared to Experiment 1. While there are not obvious explanations for this difference, one possibility is the relatively large between-subject variability that occurs in this task. The average error in the 60° FOV condition was lower in Experiment 2, but also the variability was higher in Experiment 1 (*SE* = 2.94) compared to Experiment 2 (*SE* = 1.66), suggesting that some participants with greater error may have influenced the higher mean error score in Experiment 1. As introduced earlier, we explicitly designed the vision condition manipulation to be within-subjects, so that we could assess restricted field effects despite the known individual differences in strategies used in spatial navigation learning tasks [41, 42]. For example, Furman et al. [41] identified stable biases in navigational style in individuals who used either place or response strategies during both encoding and later navigation on a virtual navigation task. Differences in navigational preferences could have influenced overall error in the experiments. In addition, since our experiments were run at different times in the academic year, it is possible that self-selection contributed to differences in strategies or that the participant sample differed in meaningful ways in terms of motivation or abilities. Furthermore, although the same building environment was used for all experiments, it varied in natural ways given the time of year (e.g., fewer people traversing hallways in the summer) which could have led to differences between the experiments. Given the importance of strategies and context especially for those with clinical low vision [43], future work should more explicitly assess individual differences in navigation strategies used under different visual restriction conditions. Work relevant to manipulating encoding and exploration strategies is currently underway in our laboratory [44] and has recently shown effects on spatial memory in older adults [45].

### Implications

Our studies have several important implications for clinical low vision. First, spatial learning through navigation with restricted peripheral field-of-view appears to be roughly comparable to having a wide FOV even down to fairly extreme restrictions. As such, clinical peripheral field loss may not negatively affect performance on some navigation tasks as long as it is not extreme, at least based on the type of spatial memory tested here. Although we did not test every possible field restriction size, participants had comparable performance to a wide FOV until the field restriction limited the FOV to foveal vision only. Second, our research also suggests that the act of mobility plays an important role in spatial memory abilities related to peripheral field loss. In our task, participants actively navigated the environment and, based on the results of the severely restricted and blind experiments, likely relied on both visual and locomotion-based information to encode the space. Future work should assess the relative contributions of active versus passive (such as being pushed in a wheelchair by someone else who is navigating) navigation in low vision survey spatial learning. It is notable that Legge et al. [6] recently found that spatial updating in a single-room space under simulated degraded viewing conditions did not differ between wheelchair and active walking conditions. Taken together, our results suggest that an effective navigation aid might aim to reduce the attentional demands involved in low vision navigation (by, perhaps, providing additional cues for obstacles or aiding in stability) but still encourage the observer to locomote as an effective form of spatial learning.

Finally, there are some limitations of extending these findings to models of clinical low vision navigation because of the use of simulated field loss. Field loss is challenging to simulate in an ecologically valid manner, as it restricts the natural eye movements in a way that would not be experienced even by an individual with some type of peripheral field loss-related low vision. However, prior research suggests similar performance on some tasks between simulated and actual peripheral field loss, although the mechanisms to achieve that performance may be different (see [4, 5]). Our research also provides useful information for performance outcomes in this task that could be compared to performance outcomes for people with clinical low vision. Additionally, we can only generalize these findings to healthy younger adults, rather than to older adults who are more likely to suffer from an extreme visual impairment, and to the survey-based pointing memory task that was used in the current paradigm. It is possible that the extent of field restriction effects may differ depending on the type of spatial learning required (e.g., route versus survey) and the complexity of the environment. While previous work suggests that attentional demands are greater for survey versus route learning, it is possible that FOV restrictions would impair tasks such as route reproduction, especially if distance perception is affected. However, the current work provides a foundation for the methodology and performance in low vision survey spatial learning while navigating, and our ongoing work with older adults, clinical low vision individuals, and different spatial tasks, aims to further address these questions of generalizability.

## Supporting Information

S1 Appendix Description of variables in dataset. (TXT)

S2 Appendix Dataset for all experiments. (XLSX)
